# The Spleen Responds to Intestinal Manipulation but Does Not Participate in the Inflammatory Response in a Mouse Model of Postoperative Ileus

**DOI:** 10.1371/journal.pone.0102211

**Published:** 2014-07-10

**Authors:** Léa M. M. Costes, Jan van der Vliet, Giovanna Farro, Gianluca Matteoli, Sjoerd H. W. van Bree, Brenda J. Olivier, Martijn A. Nolte, Guy E. Boeckxstaens, Cathy Cailotto

**Affiliations:** 1 Tytgat Institute for Liver and Intestinal Research, Academic medical center (AMC), Amsterdam, The Netherlands; 2 Adaptive Immunity Lab, Department of Hematopoiesis, Sanquin Research and Landsteiner Laboratory AMC/UvA, Amsterdam, The Netherlands; 3 Department of Clinical and Experimental Medicine, Translational Research Center for Gastrointestinal Disorders (TARGID), KU Leuven, Belgium; Charité-University Medicine Berlin, Germany

## Abstract

**Background:**

Postoperative ileus is characterized by a transient impairment of the gastrointestinal motility after abdominal surgery. The intestinal inflammation, triggered by handling of the intestine, is the main factor responsible for the prolonged dysmotility of the gastrointestinal tract. Secondary lymphoid organs of the intestine were identified as essential components in the dissemination of inflammation to the entire gastrointestinal tract also called field effect. The involvement of the spleen, however, remains unclear.

**Aim:**

In this study, we investigated whether the spleen responds to manipulation of the intestine and participates in the intestinal inflammation underlying postoperative ileus.

**Methods:**

Mice underwent Laparotomy (L) or Laparotomy followed by Intestinal Manipulation (IM). Twenty-four hours later, intestinal and colonic inflammation was assessed by QPCR and measurement of the intestinal transit was performed. Analysis of homeostatic chemokines in the spleen was performed by QPCR and splenic cell populations analysed by Flow Cytometry. Blockade of the egress of cells from the spleen was performed by administration of the Sphingosine-1-phosphate receptor 1 (S1P_1_) agonist CYM-5442 10 h after L/IM.

**Results:**

A significant decrease in splenic weight and cellularity was observed in IM mice 24 h post-surgery, a phenomenon associated with a decreased splenic expression level of the homeostatic chemokine CCL19. Splenic denervation restored the expression of CCL19 and partially prevented the reduction of splenocytes in IM mice. Treatment with CYM-5442 prevented the egress of splenocytes but did not ameliorate the intestinal inflammation underlying postoperative ileus.

**Conclusions:**

Intestinal manipulation results in two distinct phenomena: local intestinal inflammation and a decrease in splenic cellularity. The splenic response relies on an alteration of cell trafficking in the spleen and is partially regulated by the splenic nerve. The spleen however does not participate in the intestinal inflammation during POI.

## Introduction

The vast majority of patients undergoing open abdominal surgery will develop postoperative ileus (POI). POI is characterized by a transient impairment of the gastrointestinal tract leading to pain and discomfort for the patient as well as increased hospitalization costs [1]–[3]. The pathophysiology of POI relies on an inflammatory process taking place in the gut muscularis in which the activation of resident macrophages [4], [5] plays an important role. The release of pro-inflammatory cytokines such as IL-1β and IL-6 by these activated innate immune cells leads to the recruitment of leucocytes, namely neutrophils and monocytes to the gut muscularis. In turn, infiltrating leucocytes and activated resident macrophages secrete iNOS, Cox-2 and prostaglandins which are largely involved in the impairment of the gastrointestinal motility [6].

In POI, the paralysis of the gastrointestinal tract is not restricted to manipulated parts. Indeed, both the stomach and the colon are affected [7], a mechanism partially explained by the activation of neural inhibitory pathways by the local inflammation occurring in the small intestine [8]. A dissemination of the inflammation to unmanipulated parts of the gut was shown to also account for the generalized hypomotility, also referred to as “field-effect”. Enhanced pro-inflammatory cytokine and enzyme levels (i.e. IL-6, Cox2) as well as infiltration of leucocytes are observed in the colon after manipulation of the small intestine [9]. Recently, a crucial role for Th1 cells was unraveled in the dissemination of POI to the entire intestinal tract as intestinal manipulation leads to the activation of Th1 cells capable of migrating from the manipulated small intestine to the unmanipulated colon [10]. Secretion of IFNγ by these activated Th1 cells in turn triggers the activation of colonic macrophages, showing that both the adaptive and innate compartments are involved in the generalization of the ileus.

The origin of immune cells infiltrating the gut muscularis during POI remains largely unknown. However, gut associated secondary lymphoid organs were recently shown to play a role in the dissemination of the inflammation as the absence of MLN and Peyer’s patches completely abolished colonic inflammation after manipulation of the small intestine [11]. Interestingly, in other acute inflammation models namely ischemic myocardial injury, stroke and peritonitis, the population of immune cells reaching the site of inflammation (i.e. monocytes, T cells, NK cells) was shown to be released from another secondary lymphoid organ, the spleen [12]–[15]. In septic peritonitis, migration of Ly6G+CD11b+ splenic monocytes to the gut was associated with enhanced bacterial clearance and improved survival showing that the spleen can act as a cell reservoir during intestinal inflammation [15].

In light of the role of intestinal secondary lymphoid compartments in the local intestinal inflammatory process and the active role of the spleen reported during acute inflammation, we investigated whether the spleen responded to intestinal manipulation and was involved in modulating the intestinal muscular inflammation and in the pathogenesis of POI.

## Materials and Methods

### Ethical statement

All experiments were performed in accordance with the guidelines of the Laboratory Animal Use of the Netherlands and approved by the Ethical Animal Research Committee of the Academic Medical Center of Amsterdam (Protocol number: DMO 101319, DMO102498, DMO102688). All experiments were performed under fentanyl-fluanisone-midazolam (FFM) or ketamine-medetomidine-atropine (KMA) anesthesia and all efforts were made to minimize the suffering of the animals.

### Mice

Ten to 12 week-old female Balb/c were purchased from Harlan Nederland (Horst, The Netherlands) and housed with a 12/12 light/dark with *ad libitum* food and water.

### Surgical procedures and sample collection

#### Laparotomy and Intestinal Manipulation

Mice underwent Laparotomy (L) or Laparotomy followed by Intestinal Manipulation (IM) as described previously [16]. The peritoneum was opened by a midline abdominal incision and the small bowel was carefully removed from the peritoneal cavity and placed on a moist gauze pad. The entire small bowel was manipulated twice from the distal duodenum to the cecum with moist cotton applicators. Contact or stretch of the stomach or colon was avoided. Surgical procedures were performed under sterile conditions. Animals were sacrificed 24 h after L/IM.

Treatment with CYM-5442 (0.7 mg/kg) (Sigma, St Louis, MO) or vehicle injection (2% DMSO, 2% Tween20 in water) was applied i.p. 10 h after surgical procedure (Laparatomy or Laparotomy followed by Intestinal Manipulation). The injection time point was chosen according to our data showing that the loss of splenocytes begins 12 h after IM.

#### Spleen denervation

Surgical removal of the splenic nerve (Sx) or Sham operation (Sham) was performed as previously described. Briefly, the abdominal cavity was opened through a midline incision. Blood vessels irrigating the spleen were exposed and nerve bundles running along those vessels [17] were removed just before and after the first branching point of the arterial supply to the spleen using micro-surgery instruments. Two weeks later, mice underwent L or IM. This led to 4 experimental groups: *Sham L* mice underwent first Sham operation and 2 weeks later Laparotomy; *Sham IM* mice first underwent Sham operation and 2 weeks later Laparotomy followed by Intestinal Manipulation; *Sx L* mice first underwent Spleen denervation and 2 weeks later Laparotomy; *Sx IM* mice first underwent Spleen denervation and 2 weeks later Laparotomy followed by Intestinal Manipulation. Tyrosine Hydroxylase staining was performed to validate the completion of the denervation.

#### Sacrifice and samples collection

Mice were anesthetized and blood was collected by cardiac puncture. Spleens and MLN were placed in cold RPMI1640 medium (Gibco, Bleiswijk, the Netherlands) for flow cytometry analysis or snap-frozen. The entire gastrointestinal tract was placed in ice-cold oxygenated PBS (Fresenius Kabi, the Netherlands). For transcript analysis, after stripping away both mucosal and submucosal layers, colonic and intestinal muscularis segments were snap-frozen in liquid nitrogen. For MPO staining, 2 intestinal segments and the entire colon were placed in ice-cold absolute ethanol for 30 minutes and then stored in ice-cold 70% ethanol until further use.

### Measurement of the gastrointestinal transit

The gastrointestinal transit was measured using the non-absorbable tracer 70 kDa fluorescein isothiocyanate-labeled dextran (FD70) as previously described [18]. Briefly, mice were fed with 10 µL of FD70 in distilled water (6.25 mg/mL). Animals were sacrificed 90 minutes later and their entire gastrointestinal tract was placed in an oxygenated ice-cold PBS solution (Fresenius Kabi, the Netherlands) and divided into 15 segments (stomach, 10 segments of equal length for the intestine, cecum, 3 segments of equal length for the colon). FD70 concentration was assessed by fluorimetry in the supernatant of each segment. The distribution of FD70 was determined by calculation of the Geometric Center (GC) with GC = Σ(% of total fluorescent signal per segment x segment number)/100).

### Myeloperoxidase quantification by Immunohistochemistry

Muscularis of intestinal and colonic segments were stained for Myeloperoxidase (MPO) as a marker of leucocytic infiltration. Briefly, whole mount intestinal or colonic muscularis segments were stained with a 3-amino-9-ethyl carbazole (Sigma, St Louis, MO), 0.01% H_2_O_2_ in Sodium Acetate buffer (pH = 5) for 20 min, as previously described [16]. Sections were analyzed using a plain objective microscope (Zeiss Axioskop with Plan-NEOFLUAR Zeiss objectives) connected to a color-camera (JVC KY-F55 3CCD). Random counting of MPO positive cells was performed for each section, as previously described [16].

### RNA isolation, cDNA synthesis and QPCR

Total mRNAs from intestinal or colonic muscularis and spleens were extracted after homogenization of the samples in TriPure isolation reagent according to the manufacturer’s instructions (Roche Applied Science). cDNA synthesis was performed using the Revertaid first strand cDNA synthesis kit (Fermentas) and Real-time PCR was performed using a SYBR green master mix (Roche Applied Science) on a Lightcycler 480 (Roche Applied Science). The primers used (synthesized by Invitrogen, Bleiswijk, The Netherlands) are described in Table 1. Analysis was performed using the LinRegPCR program (AMC, Amsterdam, The Netherlands) [19]. The target gene expression was normalized over the expression of 2 reference genes. All data are expressed in AU and represent relative expression over the control group (i.e. L/Sham L).

**Table 1:** Primer sequences for analysis of mouse intestine and spleen samples.

	Forward primer 5′-3	Reverse primer 5′-3
HRPT	CCTAAGATGAGCGCAAGTTGAA	CCACAGGACTAGAACACCTGCTAA
Cyclophilin	ACCCATCAAACCATTCCTTCTGTA	TGAGGAAAATATGGAACCCAAAGA
Ubiquitin	AGCCCAGTGTTACCACCAAG	ACCCAAGAACAAGCACAAGG
IL-1β	CTCCTGCTGTCGGACCCAT	TGCCGTCTTTCATTACACAGGA
IL-6	GAGTTGTGCAATGGCAATTCTG	TGGTAGCATCCATCATTTCTTTGT
TNF-α	TGGAACTGGCAGAAGAGGCACT	CCATAGAACTGATGAGAGGGAGGC
CCL19	ATGCGAAGACTGCTGCC	AGCGGAAGGCTTTCACGAT
CCL21	GCTGCAAGAGAACTGAACAGACA	CGTGAACCACCCAGCTTGA
CXCL13	CATAGATVGGATTCAAGTTACGCC	TCTTGGTCCAGACACAACTTCA

### Spleen and MLN digestion and quantification

Spleens and MLN were incubated in a solution of DNaseI and Liberase TL (Roche Applied Science) in RPMI 1640 medium (Gibco, Bleiswijk, The Netherlands) or PBS respectively at 37°C for 20 min and then mechanically homogenized on a 70 µm cell strainer to obtain a single-cell suspension. The spleen and MLN cell count was determined after lysing a fraction of the cell suspension and quantified using a Coulter counter. Erythrocytes, devoid of nucleus, were therefore excluded from the count.

### FACS analysis

Spleen single-cell suspensions were incubated with a Fc-receptor blocking antibody 2.4G2 (kindly provided by Dr. Louis Boon, Bioceros BV, Utrecht, The Netherlands) and the percentage of spleen cell populations (macrophages, B cells, Dendritic cells, T cells, monocytes) were determined using the following antibodies: F4/80 (BM8) (Invitrogen, Bleiswijk, The Netherlands), CD45R (B220) (eBioscience, Vienna, Austria), CD11c (HL3) (BD Biosciences), CD4 (GK1.5) (eBioscience, Vienna, Austria), CD8α (53-6.7) (eBioscience, Vienna, Austria), CD3e (145-2C11) (eBioscience, Vienna, Austria), Lineage (Lin) (B220, NK1.1, CD90, CD49. Ly6G) (ebioscience, Vienna, Austria) CD45.1 (ebioscience, Vienna, Austria). Samples were analyzed with a LSR Fortessa II (Beckman Coulter) and the FlowJo software (Tree Star Inc., Ashland, The United States). The different spleen cell populations were defined as follow: DCs (CD11c+MHCII+), macrophages (F4/80+), B cells (CD45R+MHCII+), CD4+ (CD3+CD8-CD4+), CD8+T cells (CD3+CD8+CD4-) and monocytes (CD45+Lin-F4/80-CD11c-MHCII-CD11b+Ly-6C+). Percentages were reported to the total number of splenocytes of each mouse to calculate the number of cells per population (Figure S2 and S3).

### Cell death quantification

Apoptotic cell death was assessed using a TUNEL assay (In Situ Cell Death Detection Fluorescein kit; Roche Applied Science). Briefly, spleen sections were post-fixed in PBS PFA 4% and incubated with the Tdt enzyme diluted in digoxigenin-dUTP reaction buffer (TUNEL) for 1 h at 37°C. Sections were analyzed with a Leica fluorescence microscope and positive cells were quantified using the ImageJ software. The density of positive cells was determined as the ratio of positive cells over the area of the section.

In addition, necrotic and apoptotic cell death was determined by FACS. Spleen cell suspensions were stained with a fixable viablility Dye (eBioscience, Vienna, Austria) and Annexin V (eBioscience, Vienna, Austria) and analyzed using a LSR Fortessa II and the FlowJo software (Tree Star Inc., Ashland, The United States).

### Statistical analysis

Statistical analysis was performed using the SPSS 19.0 software (SPSS Inc, Chicago, IL). Data are expressed as mean±SEM. Normal distribution was assessed using the Kolmogorov-Smirnov test. Square-root normalization was applied to non-normal data sets. Whenever two groups of data were compared (i.e., L vs IM), a Student t-test was performed. Whenever the influence of 2 independent variables (i.e. L/IM and denervation or L/IM and CYM-5442 treatment) was analyzed, a two-way ANOVA was performed to determine the interaction between denervation (Sham *vs* Sx) and treatment (L *vs* IM). When significance was observed (i.e. p<0.05) an unpaired Student t-test was performed to evaluate the significance between Sham *vs* Sx or L *vs* IM.

## Results

### Inflammation of the gastrointestinal tract 24h after intestinal manipulation

**Figure 1:**
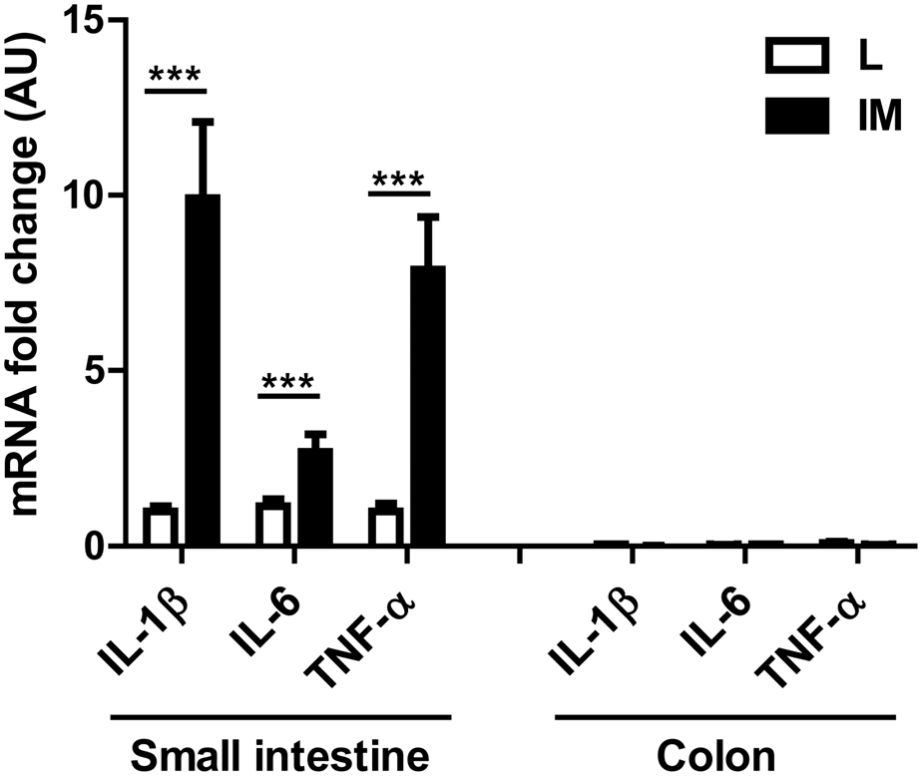
Intestinal manipulation triggers inflammation in the small intestinal muscularis but not in the colonic muscularis. Animals were sacrificed 24 after Laparotomy (L)/Intestinal Manipulation (IM). Mice undergoing L or IM display very low levels of IL-1β, IL-6 and TNF-α in their colonic muscularis compared to the levels observed in the muscularis of the small intestine. IM leads to increased mRNA levels of pro-inflammatory cytokines IL-1β, IL-6 and TNF-α in the small intestinal but not the colonic muscularis. Data shown are mean ± SEM of 3 independent experiments (n = 8–10 animals per group). ***p<0.001 (Student t-test).

As previously described, intestinal manipulation increased the expression levels of pro-inflammatory cytokines such as IL-1β (L *vs* IM: 1.00±0.24 *vs* 11.12±1.90; p<0.0001), IL-6 (L *vs* IM: 1.00±0.16 *vs* 3.28±1.06) and TNF-α (L *vs* IM: 1.00±0.16 *vs* 6.18±1.31; p = 0.0002) in the small intestine (Figure S1A and Figure 1). An increase in the number of MPO+ cells in the muscularis of the small intestine (14.60±1.60 *vs* 81.20±13.7; p = 0.011) (S1B) was also observed in manipulated mice as well as a decrease in the GC values of the gastrointestinal transit (L *vs* IM: 10.43±0.55 *vs* 4.50±0.38; p<0.0001) (Figure S1C).

As previously reported [8], [20], we did not observe any influx of MPO+ cells in the colonic muscularis after IM (data not shown). Low transcript levels of pro-inflammatory cytokines (IL-1β, IL-6 and TNF-α) were measured in the colonic muscularis and no increase in their expression was seen 24 h after IM (Figure 1).

### Secondary lymphoid organs 24h after intestinal manipulation

**Figure 2:**
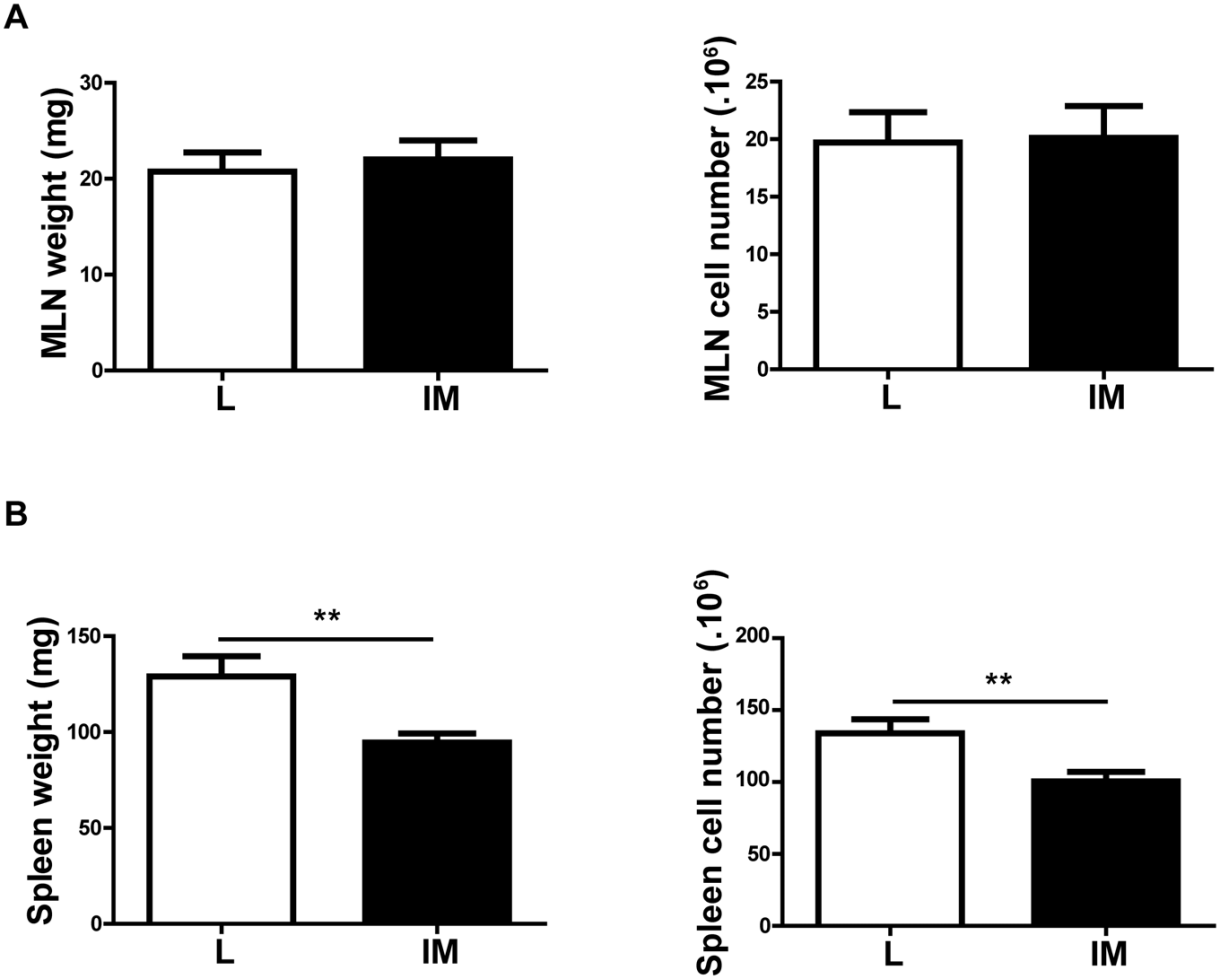
The spleen but not the MLN responds to Intestinal manipulation 24 h after surgery. (A) IM leads to a significant decrease in spleen weight and splenocyte number (B) but doesn’t affect the weight or cell number in MLN. Data shown are mean ± SEM of 3 independent experiments (n = 12 animals per group). **p<0.01 (Student t-test).

Twenty four hours after surgery, no significant difference in the MLN weight and cellularity was observed between the L and IM groups (weight: 20.73±2.00 *vs* 22.00±1.98 for L and IM mice respectively; p = 0.66; cell count: 19.72±2.62 *vs* 20.11±2.79 for L and IM mice respectively; p = 0.92) (Figure 2A).

The spleen however displayed a decrease in weight in IM compared to L mice (L *vs* IM: 129.10±10.51 *vs* 94.54±4.77; p = 0.003). This weight loss was associated with a 25% decrease in the number of splenocytes (L *vs* IM: 133.90±9.69 *vs* 100.40±6.72; p = 0.008) (Figure 2B).

### Necrotic and apoptotic cell death are not increased in the spleen upon intestinal manipulation

**Figure 3:**
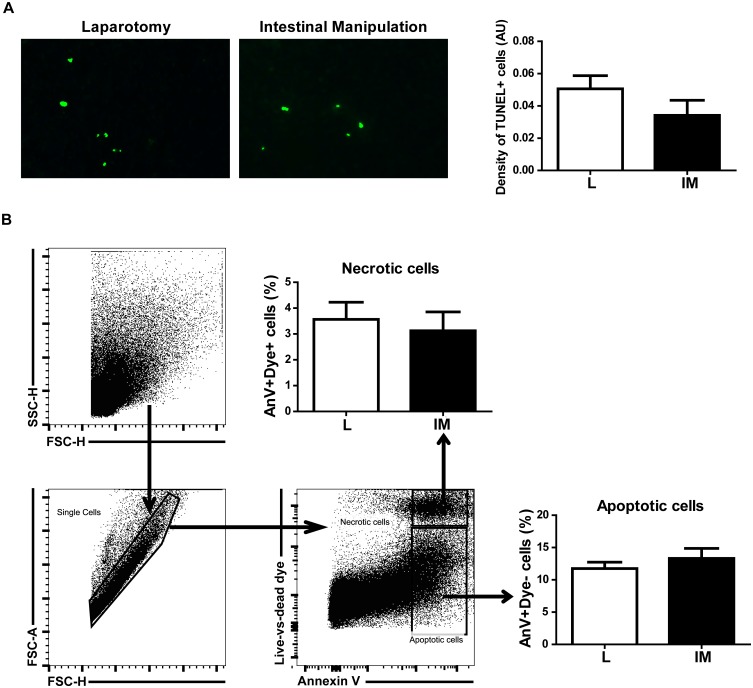
Intestinal manipulation doesn’t increase splenocyte cell death. A. Late apoptosis was determined in the spleen by a TUNEL immunofluorescent assay 24(in green) between L and IM groups. B. Necrotic (AnnexinV+ Live/dead marker+) and apoptotic cell death (AnnexinV+ Live/dead marker-) in the spleen were quantified by FACS analysis 24 h after IM. The percentage of necrotic and apoptotic cell death remains unchanged between L and IM mice. Data shown are mean ± SEM of 2 independent experiments (n = 4 animals per group) (Student t-test).

Stress related to surgical intervention can trigger a loss of splenocytes due to cell death [21]. We therefore quantified splenic cell death 24 h after surgery. No significant difference was observed in the density of cells in late apoptosis between L and IM mice (0.05±0.01 *vs* 0.03±0.01; p = 0.2) (Figure 3A). Similarly, the percentage of necrotic and apoptotic cells did not significantly differ between the L and IM groups (Necrosis: 3.56±0.66 *vs* 3.12±0.73; Apoptosis: 11.74±0.98 *vs* 13.30±1.56) (Figure 3B).

### Cell trafficking is altered in the spleen in response to intestinal manipulation

**Figure 4:**
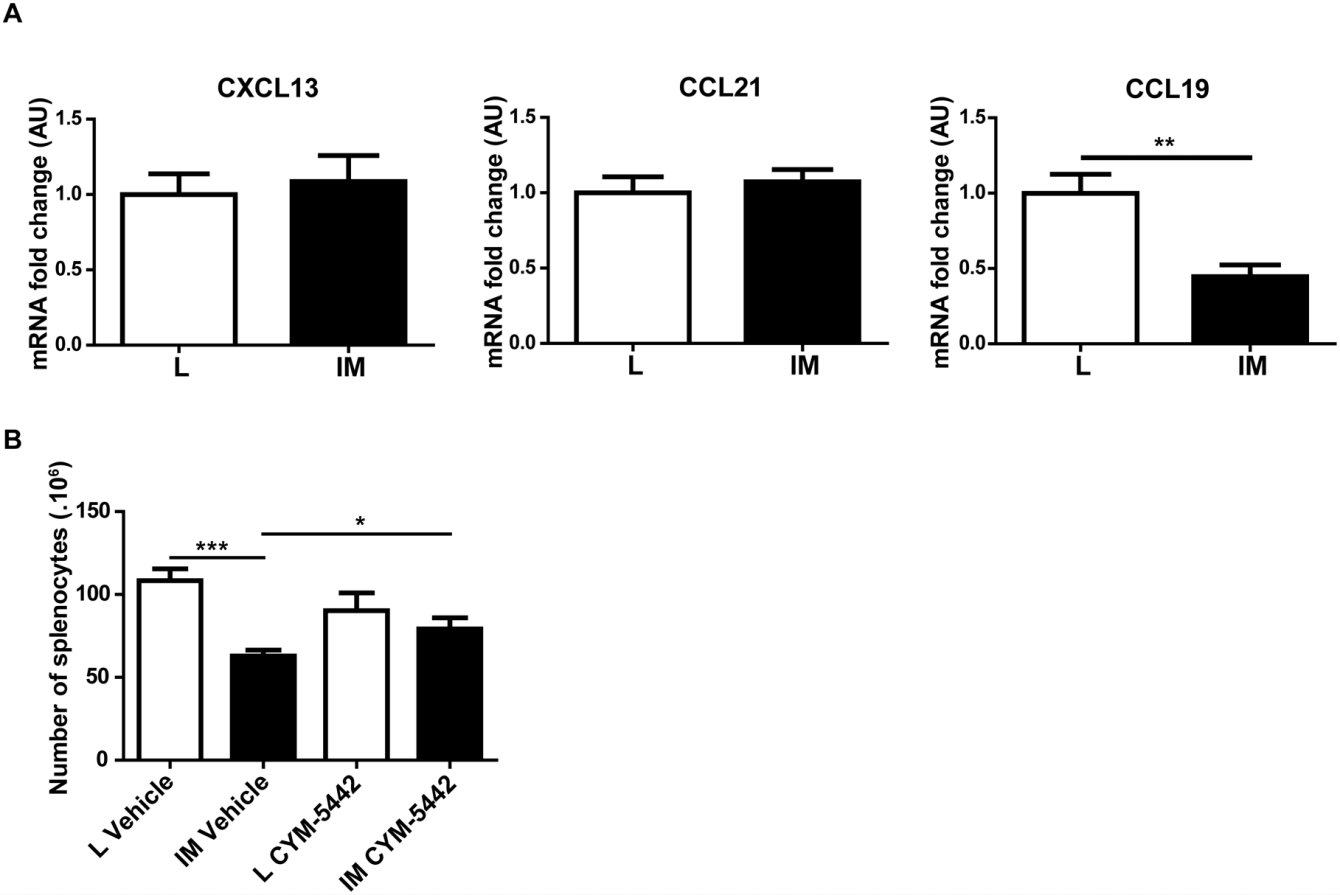
Intestinal manipulation alters cell trafficking in the spleen. (A) IM decreases the expression level of the homeostatic chemokine CCL19 in the spleen. (B) A treatment with the S1P_1_ agonist CYM-5442 (0.7 mg/kg by i.p. injection) 10 h after L/IM prevents the decrease in the number of splenocytes 24 h after IM. Data shown are mean ± SEM of 2 independent experiments (n = 8 animals per group). (A: Student t-test; B: Two-way ANOVA). *p<0.05; **p<0.01; ***p<0.001.

To investigate further the mechanism underlying the reduction in splenocytes, we analyzed the expression levels of homeostatic chemokines involved in the retention/attraction of cells to the spleen. No significant difference in the expression levels of CXCL13 or CCL21 was observed between L and IM mice. CCL19 mRNA level was however significantly lower in IM mice compared to L mice (L *vs* IM: 1.00±0.13 *vs* 0.45±0.08; p = 0.002) (Figure 4A).

To determine whether the decrease in splenocytes was due to an active departure of cells, the Sphingosine-1-phosphate receptor 1 (S1P_1_) agonist CYM-5442 [22] was injected 10 h after the surgical procedure. CYM-5442 treatment abolished the decrease in the number of splenocytes observed 24 h after IM (Figure 4B).

### Splenic innervation participates in the regulation of the egress of cells after IM

**Figure 5:**
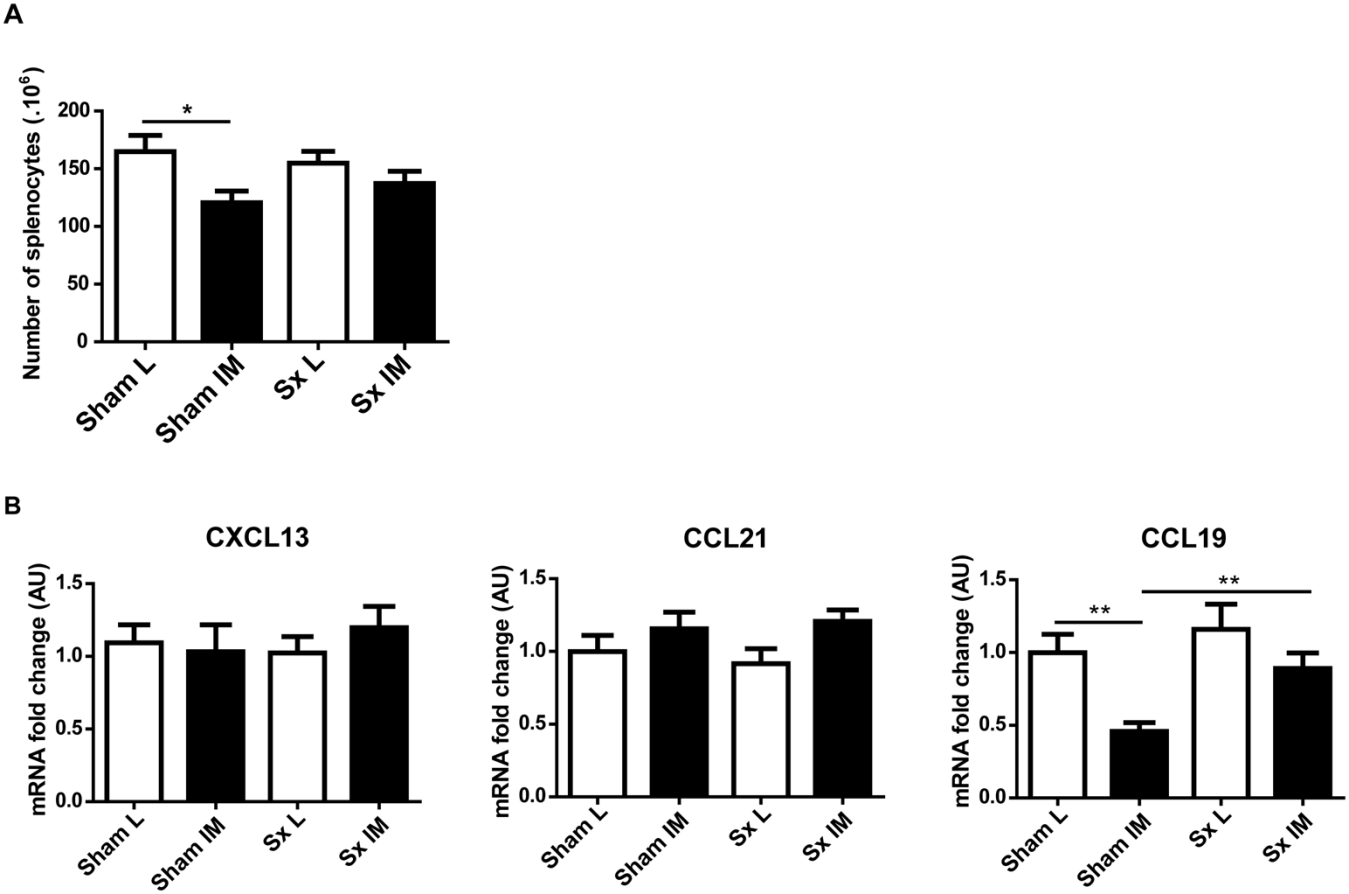
Spleen denervation prior to intestinal manipulation partially restores normal cell trafficking in the spleen. (A). Spleen denervation (Sx) partially prevents the decrease in the number of splenocytes 24 h after IM. (B) and the decrease in mRNA levels of CCL19. CXCL13 and CCL21 in the spleen were unaffected by the surgical intervention. Data shown are mean ± SEM of 3 independent experiments (n = 9–12 animals per group). *p<0.05; **p<0.01 (Two-way ANOVA).

We next addressed the role of the splenic nerve in mediating the egress of splenocytes by surgically lesioning the splenic nerve. A 25% decrease in the number of splenocytes was again observed after IM (L *vs* IM: 164.90×10^6^±13.94×10^6^ *vs* 120.60×10^6^±10.10×10^6^; p = 0.018) (Figure 5A) but IM did not induce a significant decrease in the splenocyte number in mice lacking splenic innervation (Sx L *vs* Sx IM: 154.90×10^6^±10.24×10^6^ *vs* 137.20×10^6^±10.74×10^6^; p = 0.25).

Interestingly, removal of splenic innervation prevented the decrease of CCL19 expression observed in sham-operated mice after intestinal manipulation (Sham L *vs* Sham IM: 1.00±0.13 *vs* 0.46±0.06; p = 0.003; Sx L *vs* Sx IM: 1.16±0.17 *vs* 0.89±0.11; p = 0.21; Sham IM *vs* Sx IM: 0.46±0.06 *vs* 0.89±0.11; p = 0.002) (Figure 5B).

### The number of CD4+ T cells and monocytes in the spleen is decreased 24h after intestinal manipulation

**Figure 6:**
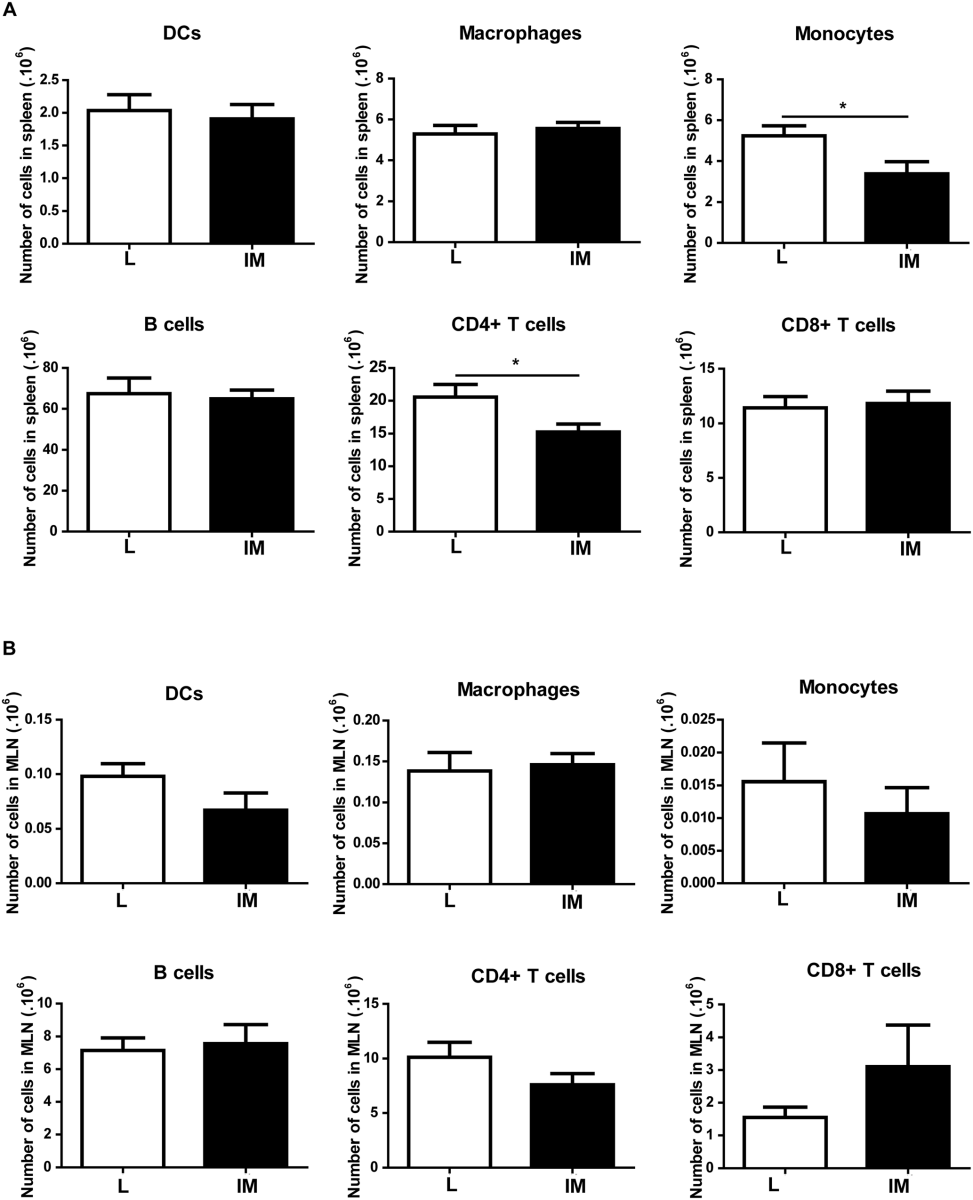
Intestinal Manipulation leads to decreased populations in the spleen but doesn’t affect the MLN. (A). The number of DCs, macrophages, B cells and CD8 T cells is not significantly altered 24 h after IM in the spleen whereas the number of CD4 T cells is significantly decreased. (B). The number of cells of all population measured remains identical in the MLN after L and IM. Data shown are mean ± SEM of 2 independent experiments (n = 8–9 animals per group). p<0.05 (Student t-test).

We then aimed to identify which population of cells was released by the spleen after intestinal manipulation by analyzing the different spleen cell populations 24 h after IM. A significant decrease in the number of CD4+ T cells was observed 24 h after IM (L *vs* IM: 20.54×10^6^±1.96×10^6^ *vs* 15.23×10^6^±1.24×10^6^; p = 0.038) (Figure 6A, S2 and S3). Similarly, the total number of monocytes was significantly decreased after IM (L *vs* IM: 5.24×10^6^±0.49×10^6^ *vs* 3.38×10^6^±0.59×10^6^; p = 0.02). No significant decrease was observed in other splenic populations (i.e., DCs, macrophages, CD8+T cells, B cells).

No change in the percentage and number of cells of the different populations measured in the MLN was observed between L and IM mice (Figure 6B, S2 and S3).

### No influx of T cells in the intestinal or colonic muscularis 24h post-IM

**Figure 7:**
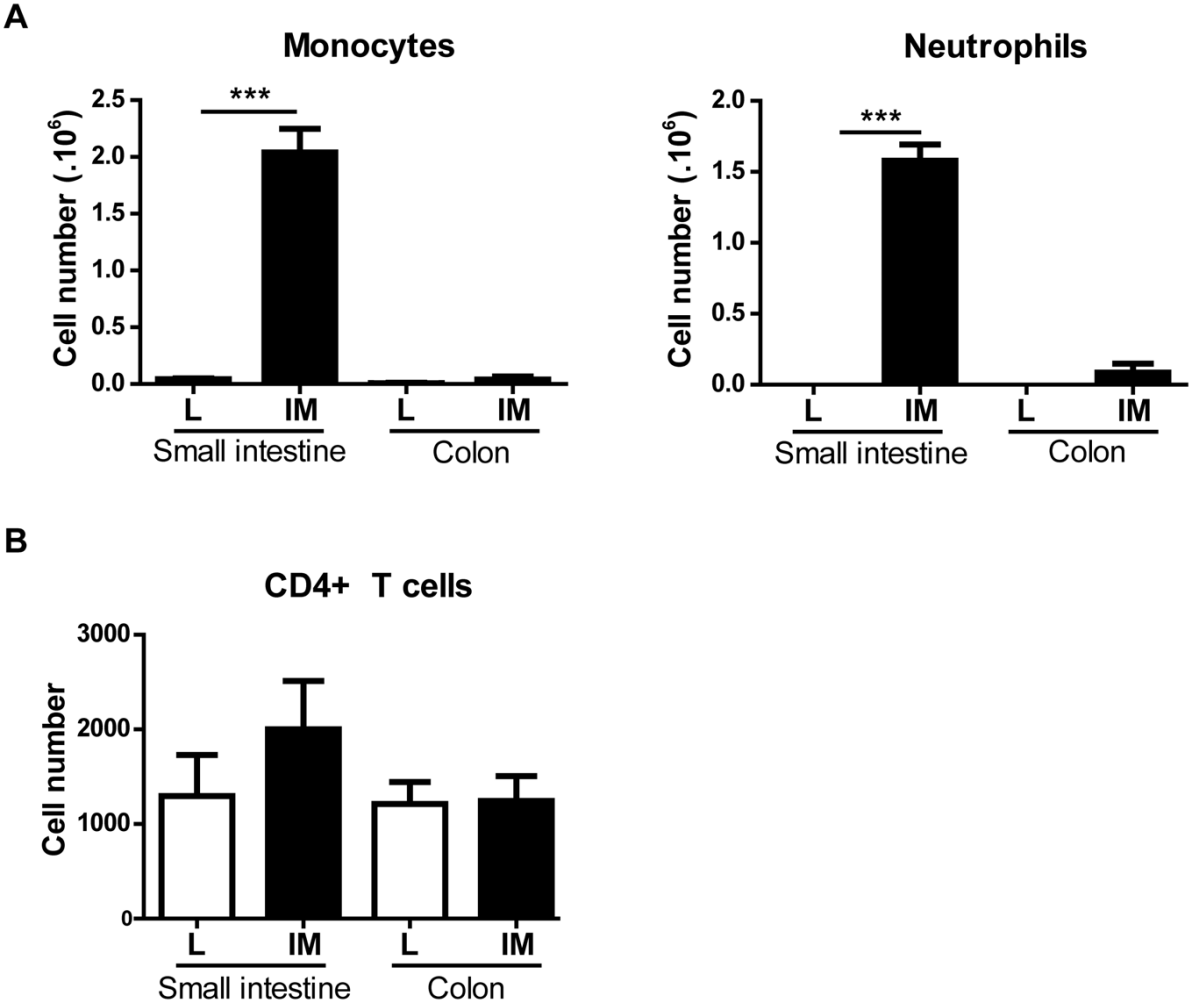
Monocytes and neutrophils are the 2 populations infiltrating the intestinal muscularis after Intestinal Manipulation. (A) Both monocytes (CD11b+Ly6G–) and neutrophils (CD11b+Ly6G+) numbers are increased in the small intestine muscularis but not the colonic muscularis 24 h after Intestinal Manipulation. (B) No influx of CD4+ T cells is observed in the muscularis of the small intestine or the colon 24 h after Intestinal Manipulation. All populations were gated from CD45+ population. Data shown are mean ± SEM of 4–5 animals per group. ***p<0.001 (Student t-test).

Next, we analyzed the cell composition in the intestinal and colonic muscularis 24 after L/IM. As previously described, immune cells infiltrating the muscularis of the small intestine 24 h after IM were mainly composed of neutrophils and monocytes (monocytes: 0.04×10^6^±0.01×10^6^ *vs* 2.04×10^6^±0.21×10^6^ for L and IM respectively; p<0.0001; neutrophils: 0.00×10^6^±0.00×10^6^ *vs* 1.58×10^6^±0.11×10^6^ for L and IM respectively; p<0.0001) (Figure 7A). The number of monocytes and neutrophils found in the colonic muscularis 24 h after IM were neglectable compared to the number of cells observed in the small intestinal muscularis (monocytes: 2.04×10^6^±0.21×10^6^ *vs* 0.04×10^6^±0.03×10^6^ for small intestine and colon respectively; neutrophils: 1.58×10^6^ *vs* 0.09×10^6^±0.06×10^6^ for small intestine and colon respectively). Moreover no significant difference in the number of these 2 populations was observed in the colon after IM (monocytes: 0.01×10^6^±0.00×10^6^ *vs* 0.04×10^6^±0.03×10^6^ for L and IM respectively; p = 0.21; neutrophils: 0.00×10^6^±0.00×10^6^ *vs* 0.09×10^6^±0.06×10^6^ for L and IM respectively; p = 0.17).

Finally, a very low number of CD4+ T cells was observed in the intestinal and colonic muscularis in L animals (1297.00±431.80 *vs* 1211.00±234.20 for small intestine and colon respectively). No influx of CD4+ T cells was observed 24 h after IM either in the intestinal (1297.00±431.80 *vs* 2000.00±512.40 for L and IM respectively; p = 0.32) or in the colonic muscularis (1211.00±234.20 *vs* 1243.00±263.40 for L and IM respectively; p = 0.93) (Figure 7B).

### The spleen does not participate in the intestinal immune response 24h post-IM

**Figure 8:**
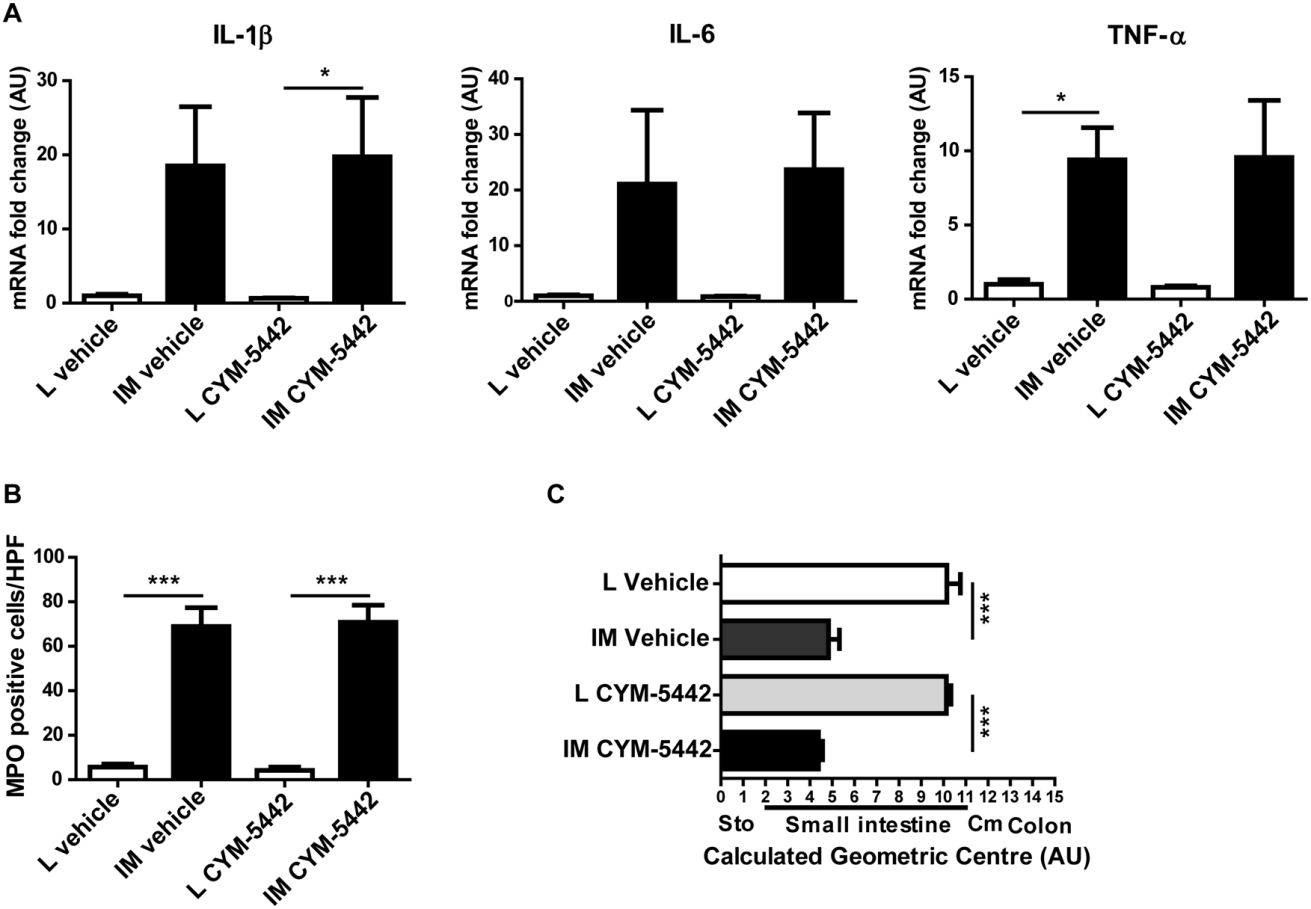
Blockade of S1P/S1P_1_ signaling doesn’t affect the inflammation or gastrointestinal transit 24 h after Intestinal Manipulation. (A) CYM-5442 treatment administered i.p.10 h after L/IM doesn’t affect the mRNA levels of IL-1β, Il-6 and TNF-α in the small intestine. (B) The number of infiltrated MPO+ cells in the intestinal muscularis 24 h after is not significantly altered in CYM-5442-treated mice. (C) Delay in the gastrointestinal transit is identical between vehicle-treated or CYM-5442-treated mice. Data shown are mean ± SEM of 2 independent experiments (n = 5–6 animals per group). *p<0.05; ***p<0.001 (Two-way ANOVA). Sto = stomach, Cm = cecum.

To assess whether the egressing splenocytes contributed to the intestinal immune response, we analyzed the inflammatory state of the intestinal and colonic muscularis in mice treated with CYM-5442. Blocking the egress of splenocytes did not affect the level of the pro-inflammatory cytokines IL-6 (21.07±13.32 *vs* 23.68±10.22 for vehicle IM and CYM-5442 IM respectively; p = 0.88), IL-1β (18.51±7.96 *vs* 19.74±8.01 for vehicle IM and CYM-5442 IM respectively; p = 0.92) or TNF-α (9.40±2.17 *vs* 9.56±3.84 for vehicle IM and CYM-5442 IM respectively; p = 0.97) (Figure 8A) in the intestinal muscularis. The influx of cells was not significantly different between vehicle- and CYM-5442-treated mice undergoing IM as shown by the MPO+ cell count in the small intestinal muscularis (68.91±8.49 *vs* 70.81±7.61; p = 0.96) (Figure 8B). The delay in gastrointestinal transit observed 24 h after IM was identical in both vehicle- and CYM-5442-treated mice (4.83±0.50 *vs* 4.39±0.18 for vehicle IM and CYM-5442 IM respectively; p = 0.37) (Figure 8C).

## Discussion

In the present study we demonstrate that intestinal manipulation leads to a response from the spleen as shown by the striking decrease in spleen weight and cellularity observed after IM. This mechanism is associated with an active egress of cells, namely CD4+ T cells and monocytes. Blocking this active departure however does not affect the severity of the intestinal inflammation after IM showing that the spleen is not involved in the intestinal inflammation and pathogenesis of postoperative ileus.

The spleen constitutes an important immune cell reservoir which can be mobilized rapidly after a trauma or at the onset of an inflammatory process [12]–[15]. Here we report that intestinal manipulation also leads to a rapid egress of cells from the spleen. We indeed observe a decrease in the number of splenocytes following manipulation of the small intestine, a phenomenon that relies on an alteration of cell trafficking to the spleen with both retention/attraction and egress signals affected during POI. The analysis of the splenic expression of homeostatic chemokines revealed a prominent decrease of CCL19 expression during POI. CCL19 acts via its receptor CCR7 to provide a homing signal to lymphoid organs for several immune cell types such as DCs, T cells [23], [24], and B cells [25]. It is therefore likely that its decreased expression observed after IM affects cell trafficking in the spleen and contributes to the decreased number of splenocytes. The sphingolipid S1P was shown to be a major modulator of cell trafficking in lymphoid organs [26]. Binding of S1P to its specific receptors expressed on immune cells triggers egress signals from lymphoid organs. Here, pharmacological blockade of this S1P/S1P_1_ axis by the administration of an S1P agonist prevented the decrease of splenocytes observed after manipulation of the intestine. Our data therefore demonstrate that manipulation of the small intestine results in an active egress of cells from the spleen through the activation of the S1P/S1P_1_ axis. We hypothesized that the egress of cells occurred consequently to intestinal inflammation rather than handling of the intestine itself. The administration of the S1P agonist was therefore performed 10 h post-surgery, a time point where intestinal inflammation has already begun as shown by the elevated levels of pro-inflammatory cytokines such as IL-1β and IL-6 in the small intestinal muscularis [20], [27].

Monocytes represent one of the major cell types infiltrating the intestinal muscularis during POI [28] but the origin of these infiltrating cells remains unclear. A recent study provided evidence of the existence of a splenic reservoir of monocytes that can rapidly egress from the spleen in case of acute inflammation [12]. Here we report that a massive and rapid egress of monocytes and CD4+T cells from the spleen occurs during postoperative ileus but that contrarily to other acute inflammation models, these cells do not participate to the intestinal inflammation. Indeed, using flow cytometry, we failed to report any major influx of CD4+ T cells to the intestinal muscularis. Similarly, preventing the egress of cells from the spleen did not ameliorate the degree of inflammation in the small intestine, influx of immune cells (neutrophils/monocytes) to the gut muscularis or delay in the gastrointestinal transit further confirming that immune cells infiltrating the gut muscularis upon intestinal manipulation do not originate from the spleen. In line, labeling of splenocytes by injection of CFSE in the spleen prior to manipulation of the intestine failed to reveal any CFSE positive cells on sections of the small intestinal muscularis 24 h after IM (data not shown). However, FACS analysis of splenocytes from injected spleens revealed that labeling of the cells was very poor as only 15% of the cells displayed positivity for CFSE positive (data not shown). Moreover, as the spleen is a highly vascularized organ, CFSE leakage from the spleen to the circulation could occur and lead to labeling of cells that didn’t egress from the spleen in response to intestinal manipulation consequently distorting the results. These technical limitations therefore prevented us to further corroborate our findings using this approach.

Of note, the pool of splenic CD4+ T cells decreased by about 5 million cells 24 h after IM while the pool of splenic monocytes decreased by about 2 million cells. Altogether, these reductions, although spectacular, cannot by themselves account for the decrease of 30 million splenocytes triggered by IM. Comparable to previous studies [29], [30], the percentage or absolute cell number of other cell types was not significantly modified after IM. Furthermore, the decrease in the number of splenocytes observed here is independent of necrotic or apoptotic cell death. This strongly suggests a general egress of splenic cell populations after IM, a phenomenon also observed during stroke [12], [29]. As blocking the S1P/S1P1 axis abolishes the decrease in the number of splenocytes, this implies that other cell populations bearing S1P receptors (i.e DCs, B cells, macrophages, CD8+ T cells) are likely to be released upon handling of the intestine. Interestingly, our data demonstrate that the sympathetic nervous system is involved in the regulation of the egress of splenocytes as shown by the absence of a significant decrease in the number of splenocytes after IM in spleen-denervated animals. Previous studies on the involvement of the splenic innervation in the trafficking of cells have reported ambivalent results. In a local inflammation model, i.e. the carrageenan air-pouch model, splenic denervation alone was not sufficient to prevent the egress of CD11b+ leucocytes from the spleen. However, in this same model, vagal nerve stimulation was shown to abolish the leucocytic egress from the spleen only when the integrity of the splenic nerve was preserved, therefore showing that the splenic innervation played an essential role [31]. On the contrary, it was recently shown that chemical sympathectomy enhances the migration of monocytes from the spleen during peritonitis [15]. In POI, splenic innervation seems to promote the egress of splenocytes out of the spleen as no significant difference is observed after IM in the number of splenocytes in spleen-denervated and sham-operated animals. Noradrenergic fibers densely innervate the T-cell zone in the spleen [32], [33], an area where stromal cells secreting homeostatic chemokines such as CCL19, CCL21 and CXCL13 are located. Neural control of the production of homeostatic chemokines by stromal cells has previously been reported in the intestine [34]. The effect of sympathetic innervation on the production of these homeostatic chemokines in the spleen is however poorly documented. Here, we demonstrate for the first time that this neural control is not restricted to the gut as splenic denervation prevents the decrease in CCL19 expression induced by IM. This suggests that sympathetic innervation of the spleen may act on stromal cells located in the T cell zone area and regulates the secretion of this homeostatic chemokine therefore playing a role in the attraction/retention of splenocytes. As noradrenalin is also known to trigger spleen contraction and atrophy [35], [36], we therefore cannot rule out that sympathetic innervation of the spleen capsule triggers a contraction of the spleen leading to a massive expulsion of cells from the spleen explaining the loss of splenocytes after IM. However, as blockade of S1P/S1P_1_ signaling abolishes the decrease in the number of splenocytes, it seems unlikely that a simple mechanical phenomenon can explain the decrease in the number of splenocytes. Importantly, no significant difference was observed in the number of splenocytes between sham-operated and spleen-denervated animals after IM showing that sympathetic splenic innervation is not the only mechanism driving this response and that other mechanisms, independent of the nervous system regulate this phenomenon.

Altogether our data suggest that the splenic response observed after IM constitutes an emergency response affecting a vast majority of splenic cell subpopulations and more strikingly CD4+ T cells and monocytes. Since our data clearly demonstrate that those cells do not participate in the intestinal inflammation underlying postoperative ileus, one may ask what the physiological relevance of this splenic response is. The influx of leukocytes to the small intestinal muscularis was previously reported to result from extravasation of cells from blood vessels to the gut wall [4]. The physiological relevance of mobilizing splenic cells rapidly and massively during POI may therefore reside in the need for the organism to maintain cellular homeostasis in the circulation by replenishing the blood cellular compartment after leucocytes are sent from the blood to the manipulated intestine. The fact that we failed to detect an effect of blockade of efflux of splenocytes on intestinal inflammation, influx of cells to the gut muscularis and delay in gastrointestinal transit further supports this concept.

In conclusion, our study demonstrates that the spleen responds to intestinal manipulation by releasing immune cells, a phenomenon associated with an alteration of cell trafficking that is partly regulated by splenic innervation. This mechanism is however not involved in the pathogenesis of postoperative ileus.

## Supporting Information

Figure S1 **Effect of Intestinal Manipulation.** Mice underwent Laparotomy (L) or Intestinal Mnaipulation (IM) and were sacrificed 24 h after surgery. IM leads to enhanced pro-inflammatory cytokine levels (i.e., IL-1β, IL-6, TNF-α) in the small intestinal muscularis (A), influx of leucocytes (B) and delayed gastrointestinal transit (C). Data shown are mean ± SEM of 3 independent experiments (n = 8–10 animals per group). *p<0.05; ***p<0.001 (Student t-test). Sto = stomach; Cm = cecum.
(TIF)

Figure S2 **Gating strategies for Flow cytometry analysis.** (A) Singlet cells were gated from the total spleen cell population. Gating on alive cells (DAPI-) was performed on singlet cells population. All populations for both spleen and MLN were gated from singlet alive cells. (B) To measure the monocytic population, a Lin-CD11b+ population was gated from singlet alive CD45+ cells. The monocytic population was determined as the F480-/MHCII-/CD11c-Ly-6C+ fraction of the Lin-CD11b+ population.
(TIF)

Figure S3 **Percentages of populations in the spleen and the MLN.** (A) Percentage of DCs (CD11c+MHCII+), macrophages (F4/80+), B cells (CD45R+MHCII+) and CD8 T cells (CD3+CD4-CD8+) increases in the spleen 24 h after IM mice whereas the percentage of CD4 T cells (CD3+CD4+CD8-) remains unchanged between L and IM mice. (B) Percentages of all populations measured in the MLN remain unchanged between mice undergoing L and mice undergoing IM.
(TIF)
