# Supplementary figures and images for: The Spleen Responds to Intestinal Manipulation but Does Not Participate in the Inflammatory Response in a Mouse Model of Postoperative Ileus
Source: PLoS One. 2014 Jul 10;9(7):e102211. doi: 10.1371/journal.pone.0102211 (PMC4092106; doi:10.1371/journal.pone.0102211)

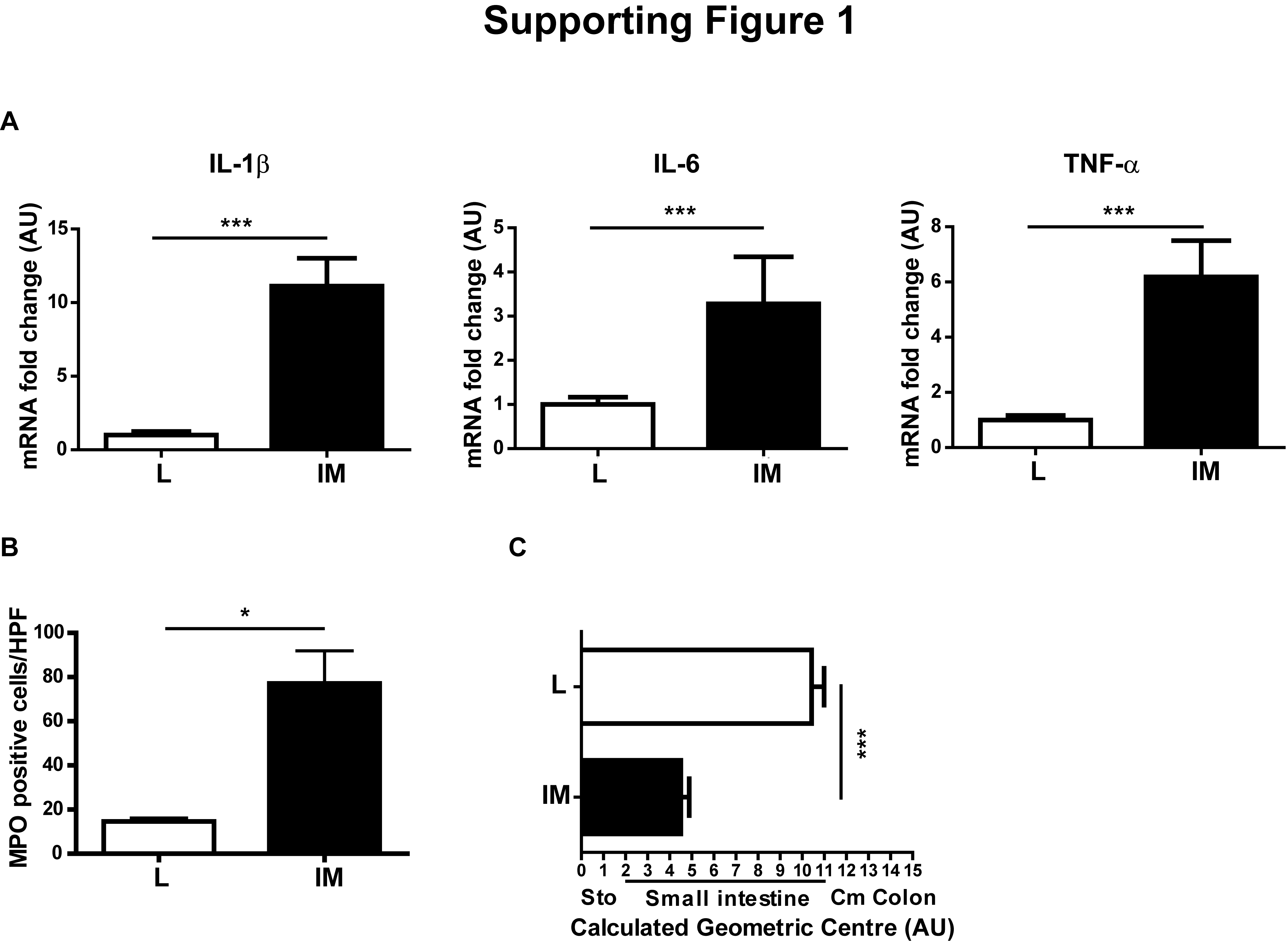

Supplement: Figure S1 — Effect of Intestinal Manipulation. Mice underwent Laparotomy (L) or Intestinal Mnaipulation (IM) and were sacrificed 24 h after surgery. IM leads to enhanced pro-inflammatory cytokine levels (i.e., IL-1β, IL-6, TNF-α) in the small intestinal muscularis (A), influx of leucocytes (B) and delayed gastrointestinal transit (C). Data shown are mean ± SEM of 3 independent experiments (n = 8–10 animals per group). *p<0.05; ***p<0.001 (Student t-test). Sto = stomach; Cm = cecum. (TIF) [file pone.0102211.s001.tif]

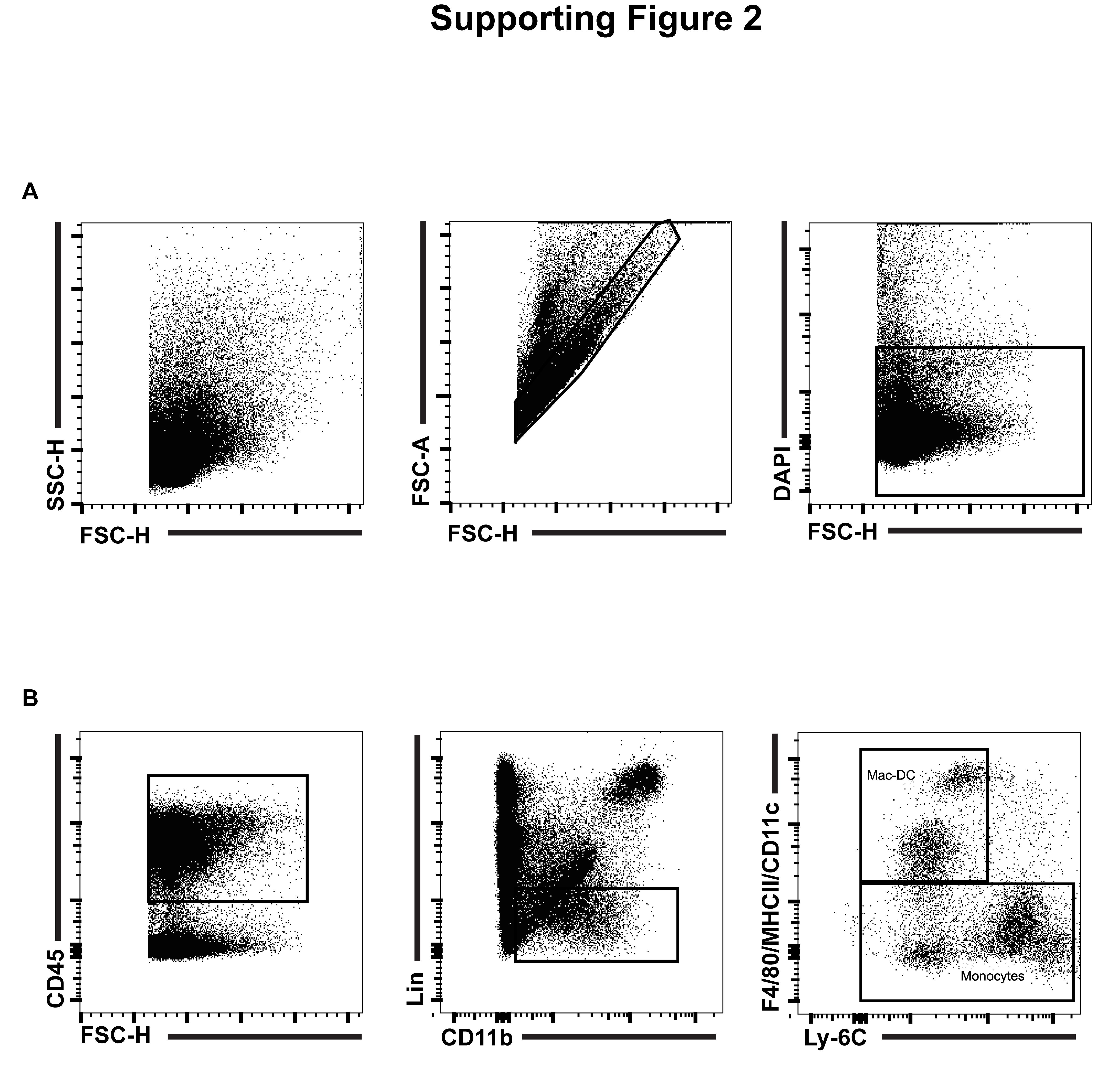

Supplement: Figure S2 — Gating strategies for Flow cytometry analysis. (A) Singlet cells were gated from the total spleen cell population. Gating on alive cells (DAPI-) was performed on singlet cells population. All populations for both spleen and MLN were gated from singlet alive cells. (B) To measure the monocytic population, a Lin-CD11b+ population was gated from singlet alive CD45+ cells. The monocytic population was determined as the F480-/MHCII-/CD11c-Ly-6C+ fraction of the Lin-CD11b+ population. (TIF) [file pone.0102211.s002.tif]

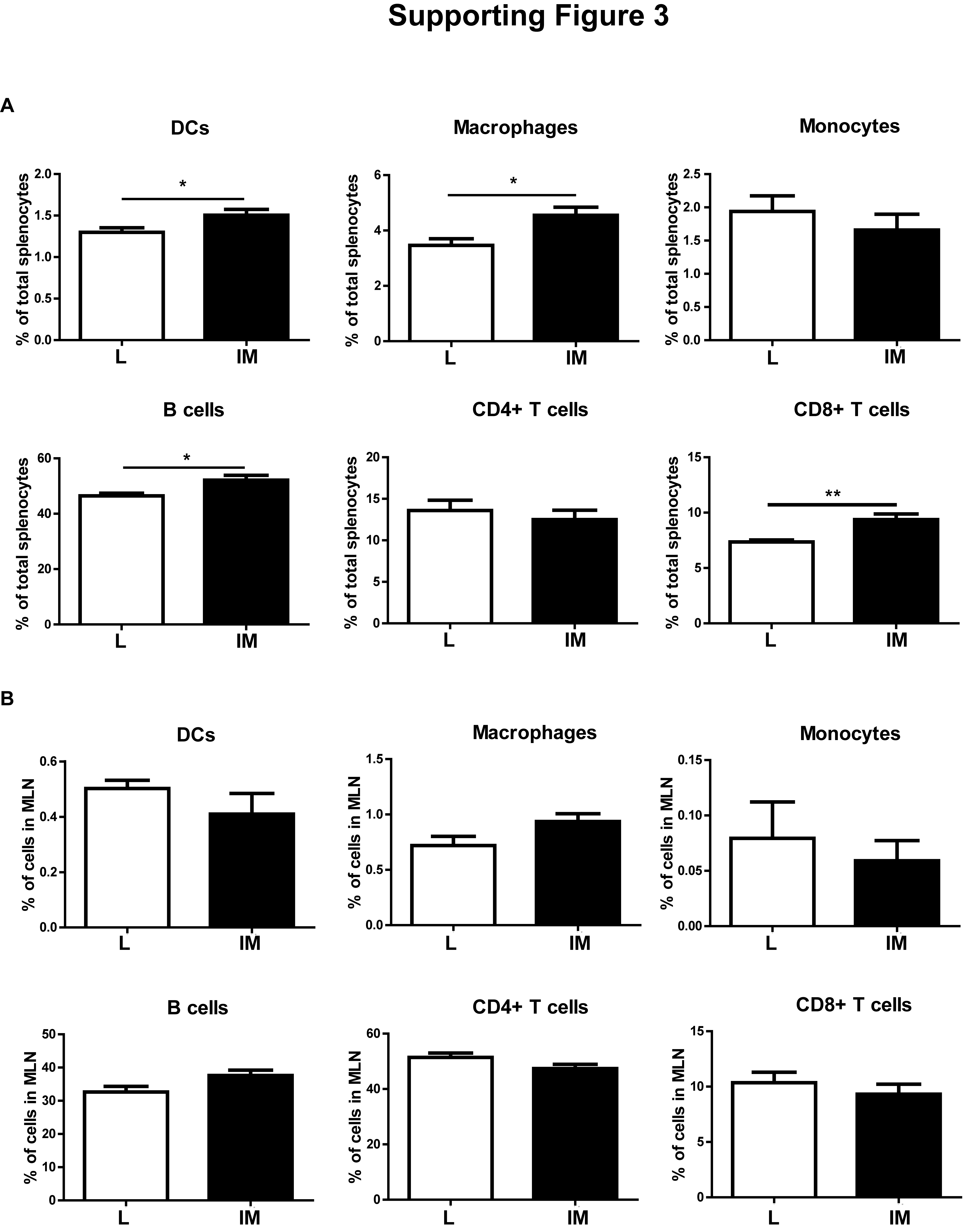

Supplement: Figure S3 — Percentages of populations in the spleen and the MLN. (A) Percentage of DCs (CD11c+MHCII+), macrophages (F4/80+), B cells (CD45R+MHCII+) and CD8 T cells (CD3+CD4-CD8+) increases in the spleen 24 h after IM mice whereas the percentage of CD4 T cells (CD3+CD4+CD8-) remains unchanged between L and IM mice. (B) Percentages of all populations measured in the MLN remain unchanged between mice undergoing L and mice undergoing IM. (TIF) [file pone.0102211.s003.tif]
